# Mutations in the TolC Periplasmic Domain Affect Substrate Specificity of the AcrAB-TolC Pump

**DOI:** 10.3389/fmolb.2020.00166

**Published:** 2020-07-21

**Authors:** Robert L. Marshall, Vassiliy N. Bavro

**Affiliations:** ^1^School of Biosciences, University of Birmingham, Birmingham, United Kingdom; ^2^School of Life Sciences, University of Essex, Colchester, United Kingdom

**Keywords:** antibiotic resistance, Gram-negative outer membrane, tripartite efflux pumps, multi-drug efflux pump, channel protein, substrate specificity, TolC, OMF

## Abstract

TolC and the other members of the outer membrane factor (OMF) family are outer membrane proteins forming trimeric channels that serve as a conduit for most actively effluxed substrates in Gram-negative bacteria by providing a key component in a multitude of tripartite efflux-pumps. Current models of tripartite pump assembly ascribe substrate selection to the inner-membrane transporter and periplasmic-adapter protein (PAP) assembly, suggesting that TolC is a passive, non-selective channel. While the membrane-embedded portion of the protein adopts a porin-like fold, the periplasmic domain of TolC presents a unique “alpha-barrel” architecture. This alpha-barrel consists of pseudo-continuous α-helices forming curved coiled-coils, whose tips form α-helical hairpins, relaxation of which results in a transition of TolC from a closed to an open-aperture state allowing effective efflux of substrates through its channel. Here, we analyzed the effects of site-directed mutations targeting the alpha-barrel of TolC, of the principal tripartite efflux-pump *Escherichia coli* AcrAB-TolC, on the activity and specificity of efflux. Live-cell functional assays with these TolC mutants revealed that positions both at the periplasmic tip of, and partway up the TolC coiled-coil alpha-barrel domain are involved in determining the functionality of the complex. We report that mutations affecting the electrostatic properties of the channel, particularly the D371V mutation, significantly impact growth even in the absence of antibiotics, causing hyper-susceptibility to all tested efflux-substrates. These results suggest that inhibition of TolC functionality is less well-tolerated than deletion of *tolC*, and such inhibition may have an antibacterial effect. Significantly and unexpectedly, we identified antibiotic-specific phenotypes associated with novel TolC mutations, suggesting that substrate specificity may not be determined solely by the transporter protein or the PAP, but may reside at least partially with the TolC-channel. Furthermore, some of the effects of mutations are difficult to reconcile with the currently prevalent tip-to-tip model of PAP-TolC interaction due to their location higher-up on the TolC alpha-barrel relative to the proposed PAP-docking sites. Taken together our results suggest a possible new role for TolC in vetting of efflux substrates, alongside its established role in tripartite complex assembly.

## Introduction

On the list of the World Health Organization’s ten priority pathogens for which new antibiotics are urgently needed, six are Gram-negative bacteria (World Health Organization, 2017). These didermic bacteria are characterized by their lipopolysaccharide-containing outer membrane, which creates a permeability barrier to decrease cytoplasmic accumulation of substances, including antibiotics, from the surrounding environment. The cytoplasmic accumulation of toxic substances is further decreased by active efflux from within the cell (Fralick and Burnskeliher, 1994; Nikaido, 1994; Krishnamoorthy et al., 2017; Westfall et al., 2017). Antibiotic efflux systems therefore represent attractive targets in overcoming antibiotic resistance.

**FIGURE 1:**
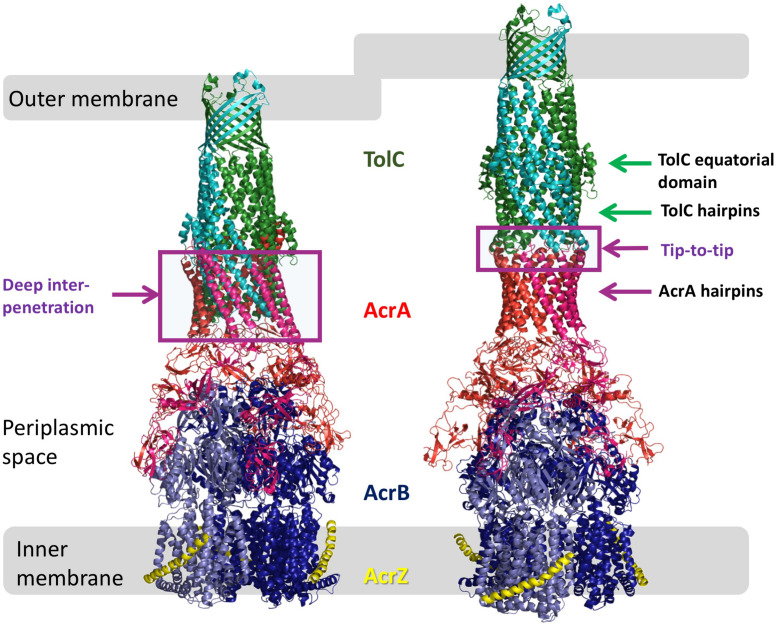
Models of AcrAB-TolC interaction. Comparison of the OMF-PAP interaction in “deep-interpenetration” models (left) and the “tip-to-tip” models (right) derived from the experimental cryo-EM structures of AcrABZ-TolC (based on 5O66.pdb; Wang et al., 2017).

In *Escherichia coli*, the tripartite pump AcrAB-TolC is principally responsible for efflux-mediated multidrug resistance (Nishino and Yamaguchi, 2001; Sulavik et al., 2001). It consists of the RND-family transporter AcrB, the outer membrane-embedded channel-protein TolC and, bridging the two (Figure 1), the periplasmic adapter protein (PAP) family member, AcrA (Misra and Bavro, 2009; Symmons et al., 2015; Du et al., 2018). Deletion of the gene encoding any one of these three proteins from the chromosome increases susceptibility of *E. coli* to many antibiotics (Husain and Nikaido, 2010), as does mutational inactivation of AcrB or chemical inhibition of AcrB efflux activity (Husain and Nikaido, 2010; Opperman et al., 2014).

Each individual protein is suggested to play a defined role within the tripartite assembly. The Resistance-nodulation-division (RND) transporter AcrB is an inner membrane protein that forms a homotrimer and has extensive periplasmic domains that are thought to be responsible for drug binding and transport, and interaction with partner proteins (Elkins and Nikaido, 2002; Murakami et al., 2002). Transport itself is driven by conformational changes induced by proton translocation through the transmembrane domain (Murakami et al., 2006; Seeger et al., 2006; Eicher et al., 2014).

The energized inner membrane RND-transporter AcrB is linked to the outer membrane-bound TolC by the PAP AcrA. AcrA is a multidomain periplasmic protein, with a hairpin-like topology, with both N- and C-terminal ends of its polypeptide chain contributing to each of the 4 principal domains, which are arranged in a string-like fashion. The membrane proximal (MPD) domain alongside the neighboring β-barrel and lipoyl domains are thought to interact with AcrB (Du et al., 2014; McNeil et al., 2019), while an α-helical coiled-coil hairpin domain forms an extension, which in all models of assembly extends beyond AcrB further into the periplasm allowing AcrA to interact with TolC via an as-yet unconfirmed interface (Mikolosko et al., 2006; Stegmeier et al., 2006; Lobedanz et al., 2007; Symmons et al., 2009; Du et al., 2014; Daury et al., 2016; Shi et al., 2019).

Current consensus suggests that TolC provides a gated, but otherwise passive channel (Zgurskaya et al., 2011), which is highly promiscuous and capable of transporting equally well small drugs and megadalton-sized repeats-in-toxin (RTX) adhesins across the outer membrane (Smith et al., 2018).

**FIGURE 2:**
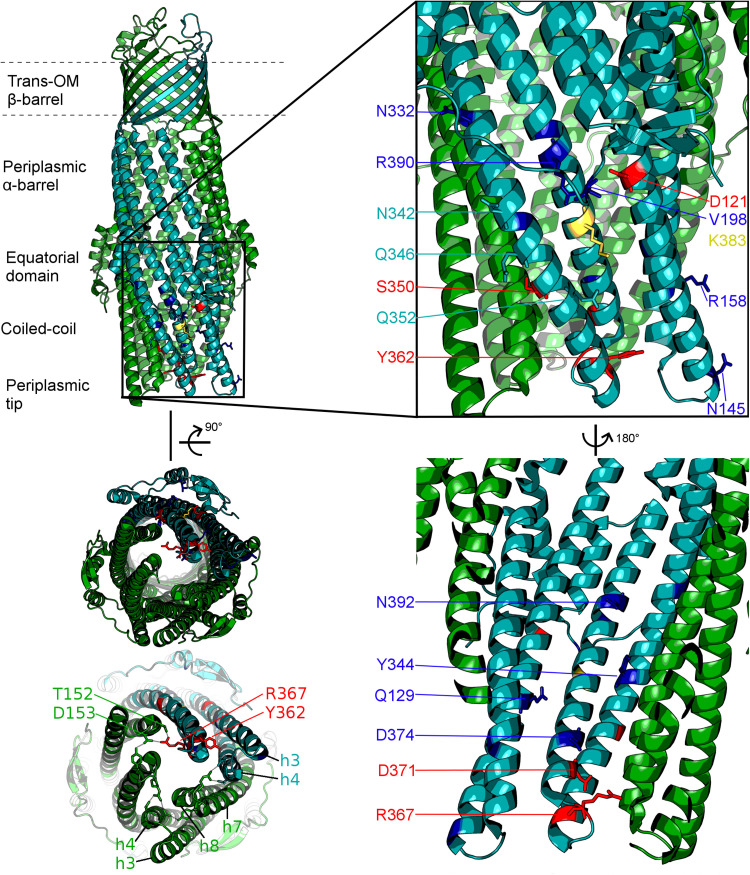
Localisation of the mutated residues discussed within the context of the TolC structure. Onto a single protomer of TolC (1EK9, teal) are mapped the positions mutated in the current study, shown in stick format and color-coded according to the effect of the respective mutation as determined by growth kinetics assays. Note that, mutations that have a substrate-specific effect are not localized to a specific region of the TolC α-barrel. Blue: mutation has an antibiotic-specific effect. Red: mutation presents same phenotype as BW*ΔtolC*. Yellow: effect observed is dependent on the substitution. Residues colored in teal: mutation has no effect. Helices belonging to the adjacent protomers are colored green. Lower-left panel shows the view of the TolC channel from the periplasmic aperture side, with helices forming the mobile (H7/H8) and static (H3/H4) hairpins being labeled, alongside with the key gating residues T152, D153, Y362, and R367.

The TolC from *E. coli* is the prototypical representative of the outer-membrane factor (OMF) family (Paulsen et al., 1997), which is widely spread within Gram-negative bacteria, and also includes OprM from *Pseudomonas aeruginosa*, VceC from *Vibrio cholerae* and MtrE from *Neisseria gonorrhoeae* amongst others (Federici et al., 2005; Janganan et al., 2011; Zgurskaya et al., 2011; Monlezun et al., 2015; Phan et al., 2015). All of these are homotrimeric outer membrane proteins consisting of a transmembrane β-barrel, an extended discontinuous periplasmic coiled-coil domain and a mixed α-helical/β-stranded equatorial domain of unknown function (Koronakis et al., 2000; Federici et al., 2005; Monlezun et al., 2015). Together with the transmembrane β-barrel, the coiled-coil domain (also known as the α-barrel) of TolC forms a pseudo-continuous tube that is occluded at the periplasmic end by converging tips of the helical hairpins formed by helices H3/H4 and H7/H8 (sometimes referred to as the TolC-hairpin domain). The tips of these hairpins are held together by a set of strong ion-bridge interactions, involving residues T152, D153, Y362, and R367 (see Figure 2). These interactions can be disrupted by mutagenesis resulting in a spontaneous relaxation of the helical-hairpin trajectories of the so-called “mobile” helices H7/H8 relative to the “static” H3/H4 hairpins leading to TolC channel opening (Andersen et al., 2002a; Bavro et al., 2008; Pei et al., 2011). Under physiological conditions these interactions are speculated to be unlocked by interaction with the cognate PAP (in this case AcrA), and are hence referred to as “primary gates” (Bavro et al., 2008; Janganan et al., 2013). While the exact mechanism is still a matter of debate, there are two prevailing models of OMF-PAP interaction (Symmons et al., 2015). The first model is primarily based upon biochemical data and involves AcrA-hairpin domain wrapping around the lower portion of the TolC α-barrel, fitting into the so called inter-protomer and intra-protomer helical grooves formed by the H3/H4 and H7/H8 hairpins in a deeply interpenetrative fashion forming inter-protein helical bundles with the TolC hairpins (Fernandez-Recio et al., 2004; Stegmeier et al., 2006; Lobedanz et al., 2007; Tikhonova et al., 2011). The second model is derived predominantly from cryo-EM structural data suggesting only a limited interface between the AcrA hairpins and the tips of the TolC coiled-coils hence it is called the “tip-to-tip” model of interaction (Xu et al., 2011), and results in an extended uniform tube (Du et al., 2014; Kim et al., 2015; Daury et al., 2016; Shi et al., 2019; Figure 1). While most of the recent cryo-EM (Kim et al., 2015; Daury et al., 2016; Wang et al., 2017; Tsutsumi et al., 2019) and cryo-tomography structures (Shi et al., 2019) have favored the tip-to-tip model, there is still some ambiguity whether deeper interpenetration may take place transiently during part of the efflux-cycle (Hayashi et al., 2016).

It has long been hypothesized that the specificity to efflux substrates resides solely within the energized RND-pump component of the tripartite assembly (Elkins and Nikaido, 2002; Middlemiss and Poole, 2004). Indeed, a number of crystallographic (Nakashima et al., 2011, 2013; Eicher et al., 2012) and mutagenesis studies (Middlemiss and Poole, 2004; Husain et al., 2011; Soparkar et al., 2015) have suggested that drug selectivity resides within the large cavities of the AcrB transporter, namely the so-called “proximal” and “distal” binding pockets, which are separated by the drug-vetting switch-loop (Nakashima et al., 2011, 2013; Eicher et al., 2012), as well as by the interprotomer vestibules (Husain et al., 2011). Furthermore, adaptive mutations selected under direct antibiotic pressure have been shown to affect not only general regulators of efflux pump gene expression, but also to specifically affect the drug binding pocket of AcrB and related transporters (Blair et al., 2015; Yao et al., 2016), altering the profile of exported drugs.

The above data points strongly to the primacy of the AcrB (and RND-transporters in general) in the selection of the substrates within the tripartite multidrug efflux-assemblies. While the role of PAPs in substrate selection is less clear, several studies have highlighted their possible involvement in the case of metal-transporting RND-pumps (De Angelis et al., 2010; Chacon et al., 2014) and substrate-dependent activation of both ABC- and RND-transporter based tripartite assemblies (Tikhonova et al., 2007; Lin et al., 2009; Verchere et al., 2015). Adding to the complexity, there is also a notable redundancy amongst PAPs, which arises from their promiscuous interaction with a number of RND-partner pumps, allowing for added resilience and an ability to export similar, but different substrates (Smith and Blair, 2014; McNeil et al., 2019).

In comparison to the transporters and PAPs discussed above, the role of TolC in selection and vetting of the export cargoes has remained relatively unexplored. To our knowledge, no conclusive substrate-specific role has been reported for TolC and the channel is seen as a passive conduit in terms of efflux specificity. To address this gap in our understanding of the role of OMFs in relation to substrate selection and to clarify the mode of their possible association with its cognate PAPs, in the present study, we analyzed the predicted solvent-exposed residues of the lower-portion of the TolC α-barrel, which are likely to engage the potential PAP-hairpins under both *“deep-interpenetration”* and *“tip-to-tip”* interaction scenarios discussed above. We then targeted these using site-directed mutagenesis and performed functional assays on the resulting mutants to evaluate their effect on the pump activity and substrate selectivity using a range of clinically relevant antibiotics.

Here, we identify mutations targeting residues of the coiled-coil interface of TolC α-barrel that impact not just overall efficiency of efflux, but also alter the specificity of the pump to several compounds, highlighting a previously underappreciated role of the OMF channels in vetting the efflux substrates.

## Materials and Methods

### Strains and Plasmids

The kanamycin resistance marker was removed from the BW25113 *tolC::aph* Keio strain JW5503 (Baba et al., 2006), using the pCP20 protocol (Cherepanov and Wackernagel, 1995; Datsenko and Wanner, 2000), to generate BW*ΔtolC*. In order to clone the *tolC* gene and for subsequent mutagenesis we generated a custom plasmid pASK-RM, which was produced by three rounds of mutagenesis on pASK-IBA13 (IBA Technologies), as described in the Supplementary Information. The pASK-*tolC* plasmid was made by cloning *tolC* amplified from the MG1655 chromosome into pASK-RM using the NdeI and XhoI restriction sites. All mutations were made on the pASK-*tolC* plasmid using the Quikchange Lightning site-directed mutagenesis kit (Agilent), in accordance with the manufacturer’s instructions. Both the BW25113 wild-type Keio parent strain and the BW*ΔtolC* derivative were transformed with pASK-RM using the Transformation and Storage Solution (TSS) method (Chung et al., 1989); BW*ΔtolC* was transformed with pASK-*tolC* and the mutated plasmids using the TSS method. All plasmid-containing strains were maintained on Lennox agar supplemented with 100 μg.ml^–1^ ampicillin, and cultured in Lennox broth supplemented with 100 μg.ml^–1^ ampicillin.

### Determination of Minimum Inhibitory Concentrations (MICs)

All MIC assays were designed to comply with standards set out by the British Society of Antimicrobial Chemotherapy for MIC determination by agar dilution (Andrews, 2012). In modification to the standard method, anhydrotetracycline was added to cooled molten Iso-Sensitest agar (Oxoid) to a final concentration of 20 ng.ml^–1^ before dispensing.

### Growth Kinetics Assays

To Iso-Sensitest broth (Oxoid), anhydrotetracycline was added to a final concentration of 2 ng.ml^–1^ and the test antibiotic to the final concentration as required. Single colonies were inoculated into 2 ml Lennox broth supplemented with 100 μg.ml^–1^ ampicillin and 2 ng.ml^–1^ anhydrotetracycline, and incubated at 37°C with shaking at 200 rpm for 8 h. Bacteria were harvested by centrifugation (21,000 × *g* for 5 min) and resuspended to 1/200 the original cell density in Iso-Sensitest broth supplemented with 2 ng.ml^–1^ anhydrotetracycline. In 96-well plates, 200 μl of the broth-anhydrotetracycline-test antibiotic solution was inoculated with 2 μl of the bacterial suspension. Growth was measured on a Fluostar Optima (BMG Biotech) or Fluostar Omega (BMG Biotech), at 37°C with orbital shaking at 200 rpm between read cycles. The OD_600_ was measured every 3 min for 8 h using 10 flashes per well per read. The generation time was calculated from the growth curves, and normalized to the BW*ΔtolC* pASK*-tolC* strain in the same conditions on the same plate to give a relative growth rate.

### Dye Efflux Assays

Efflux assays using Nile Red as the substrate were modified from previously published methods (Bohnert et al., 2010; Iyer et al., 2015). Overnight cultures were diluted 1:1000 in Lennox broth supplemented with 100 μg.ml^–1^ ampicillin and 2 ng.ml^–1^ anhydrotetracycline, and incubated at 37°C with shaking at 180 rpm for 2 h to reach early exponential phase. Cells were harvested from 1 ml of this culture by centrifugation at 21,000 × *g* for 5 min at room temperature and resuspended in phosphate buffered saline (PBS) to a volume in milliliters equal to the culture OD_600_. Carbonyl cyanide m-chlorophenyl hydrazone (CCCP) and Nile Red were added to 150 μl of the cell suspensions at final concentrations of 100 μM and 5 μM, respectively, and incubated for 1 h at room temperature. To remove free Nile Red and CCCP, cells were harvested (21,000 × *g* for 5 min at room temperature) and resuspended in 150 μl fresh PBS. To wells on a black-sided tissue-culture treated 96-well plate (4titude), 150 μl PBS and 25 μl pre-loaded cells were added. The OD_600_ was measured once on a Fluostar Optima, before the fluorescence was measured with excitation at 550 nm and emission at 640 nm with 27 s between readings. Fluorescence was measured for 5 readings before injection of 25 μl 70 mM glucose and continued for 25 readings post-injection.

### Membrane Protein Analysis

Overnight cultures were diluted 1:1000 in 500 ml Lennox broth supplemented with 100 μg.ml^–1^ ampicillin and 2 ng.ml^–1^ anhydrotetracycline, and incubated at 37°C with shaking at 180 rpm for 3 h to reach mid exponential phase. Cells were harvested by centrifugation at 4,000 × *g* for 20 min, resuspended in 30 ml resuspension buffer (20 mM Tris–HCl pH8.0, 200 mM NaCl) and lysed on an Emulsiflex C3 homogeniser. Cell debris were removed by centrifugation at 8,000 × *g* for 20 min at 4°C and membranes were harvested from the supernatant by centrifugation at 100,000 × *g* for 1 h at 4°C. Purified membranes were homogenized in 2 ml Tris/glycerol solution (20 mM Tris–HCl pH6.8, 10% v/v glycerol) and solubilised by addition of 1% n-octyl-β-D-glucopyranoside. Protein concentrations were determined by Bradford assay in a microtitre plate. An equal amount of total protein for each sample was resolved by SDS-PAGE on 10% gels. Proteins were transferred to nitrocellulose membranes using the iBlot dry transfer system. Membranes were probed with rabbit-raised anti-TolC polyclonal antibody (kindly provided by Helen Zgurskaya, Oklahoma State University) and anti-rabbit alkaline phosphatase-conjugated secondary antibody. The blot was developed with the chromogenic AP-substrate BCIP/NBT (Abcam).

## Results

In this study, we targeted residues that are solvent-exposed on the surface of the α-barrel of TolC and its helical hairpins (H3/H4 and H7/H8), based on the available X-ray structures of TolC (Koronakis et al., 2000; Bavro et al., 2008; Pei et al., 2011), to establish their potential impact on the efflux and drug susceptibilities of the resulting mutant pumps (Figure 2 and Supplementary Table 1). For historical consistency the numbering of the TolC residues used in the text below is the accepted one from the PDB entry 1EK9 (Koronakis et al., 2000) and corresponds to the mature protein lacking the signal sequence. A full description of the logic of the residue selection is provided in the “Discussion” section below and in Supplementary Material).

### Minimum Inhibitory Concentration Tests Identify TolC Mutations Causing Selective Substrate-Sensitivity and Vancomycin Sensitivity

A library of mutations in *tolC* was made on the pASK-*tolC* plasmid (see Table 1 for a full-list and Supplementary Information for the description of location of each mutation). As the *tolC* gene was under the control of the tetracycline promoter, a simple broth MIC assay was used to determine a suitable concentration of the inducer, anhydrotetracycline (Gossen and Bujard, 1993), to add for functional assays (data not shown). As both tested concentrations of anhydrotetracycline (2 and 20 ng.ml^–1^) caused the MICs of erythromycin and fusidic acid to be the same for BWΔ*tolC* pASK-*tolC* as for BW25113, it was decided that anhydrotetracycline should be added at 2 ng.ml^–1^ for functional assays in broth and at 20 ng.ml^–1^ for MIC assays on agar.

**TABLE 1:** Minimum inhibitory concentrations for the TolC mutants.

**Strain**	**Acf**	**Cam**	**Doc**	**Fus**	**Nal**	**Nov**	**Tet**	**Van**
BW25113 pASK-RM	64	4	4096	512	8	512	1	256
BW*ΔtolC* pASK-RM	**4**	**0.5**	**128**	**4**	**1**	**4**	0.25	256
BW*ΔtolC* pASK-*tolC*	32	2	4096	64	4	512	0.5	256
D121N	16	2	4096	128	2	512	0.25	256
Q129L	32	2	4096	64	2	512	**0.125**	128
N145L	**8**	1	4096	32	**0.5**	512	**0.125**	128
R158D	32	2	4096	64	4	512	0.25	128
V198D	32	2	4096	64	4	512	0.25	128
N332L	32	2	4096	128	4	512	0.25	256
N342A	16	2	4096	64	4	512	0.25	256
Y344F	32	2	4096	64	2	512	**0.125**	**64**
Q346L	32	2	4096	64	2	512	0.25	256
S350F	**2**	**0.25**	**128**	**2**	**0.5**	**2**	0.25	256
Q352A	32	2	4096	64	4	512	0.25	**64**
Q352E	**8**	2	4096	64	2	256	0.25	256
YFRS	**4**	**0.5**	**256**	**4**	**0.5**	**4**	0.25	256
D371V	**1**	**0.25**	**64**	**1**	**0.5**	**1**	**0.125**	256
D374V	**8**	2	4096	64	2	512	0.25	256
K383D	**8**	2	**512**	64	**0.5**	512	**0.125**	128
K383E	16	2	4096	128	4	512	0.25	256
R390E	32	2	4096	64	4	512	0.5	256
RENT	32	2	4096	32	2	512	**0.125**	**32**

*Minimum inhibitory concentrations in μg.ml^–1^ of acriflavine (Acf), chloramphenicol (Cam), deoxycholic acid (Doc), fusidic acid (Fus), nalidixic acid (Nal), novobiocin (Nov), tetracycline (Tet) and vancomycin (Van) against the library of mutants. Bold indicates values significantly different to the complemented strain (≥2 doubling dilutions difference). Additional visualization of the data presented in provided in Supplementary Figure 2.*

Minimum inhibitory concentrations of a variety of substances against the library of *tolC* mutations were determined using the agar dilution method (Table 1 and Supplementary Figure 2). Of the substances tested, all except vancomycin are reportedly substrates of AcrAB-TolC (Nishino and Yamaguchi, 2001; Sulavik et al., 2001). Vancomycin is commonly used to report on the integrity of the outer membrane permeability barrier, as the OM is normally impenetrable for it, due to its high molecular weight and inability to pass *via* either TolC or constitutively expressed porins. Therefore vancomycin susceptibility gives an indication as to how open or closed the TolC channel may be (Bavro et al., 2008).

Three mutations (Y344F, Q352A and the R390E/N392T “RENT” double mutant) caused markedly increased susceptibility to vancomycin, suggesting that these mutations cause disruption of the outer membrane permeability barrier at the level of TolC gating. Of these three mutations, Q352A did not change susceptibility to any of the other tested substances; while both Y344F and RENT increased susceptibility to tetracycline in addition to vancomycin. Notably, while the RENT double mutant increased susceptibility to vancomycin and tetracycline, the R390E single mutant did not have any effect on the MIC of any tested substance. Seven other mutations (D121N, R158D, V198D, N332L, N342A, Q346L, and K383E) also had no effect as determined by MIC testing.

Consistent with prior results (Schuster et al., 2016), in this study, the MIC of rifampicin against both BW25113 pASK-RM and BW*ΔtolC* pASK-RM were the same (data not shown), indicating that it is not a substrate of the AcrAB-TolC. Having excluded rifampicin, the D371V mutation caused increased susceptibility to all of the substrates tested. It was also observed that this mutation caused a decreased growth rate both in broth and on agar, colonies and cultures were paler in color than any of the other strains tested, and colonies were more mucoid with less well-defined edges.

Of the remaining mutations, each increased susceptibility to only a single substance – namely tetracycline (Q129L) and acriflavine (Q352E and D374V). Mutations N145L and K383D increased susceptibility to acriflavine, nalidixic acid and tetracycline; while the K383D mutation additionally increased susceptibility to deoxycholic acid. Meanwhile, and consistent with prior reports (Augustus et al., 2004; Pei et al., 2011) both S350F, and the Y362F/R367S (YFRS) double-mutant increased susceptibility to all tested substrates except tetracycline.

### Growth Kinetics Assays Reveal Substrate-Specific Effects of the Mutations

As MIC assays give only an end-point perspective on the growth of a strain in the presence of antibiotics, growth kinetics assays were used to derive finer detail regarding the effects of mutations. Each strain was grown in Iso-Sensitest broth supplemented with 2 ng.ml^–1^ anhydrotetracycline and either: no antibiotic, 500 μg.ml-1 deoxycholic acid, 1 μg.ml-1 nalidixic acid, 1 μg.ml-1 chloramphenicol, 200 μg.ml-1 fusidic acid or 100 μg.ml-1 vancomycin. Growth rates in exponential phase were calculated as generation time in minutes, and normalized as a percentage of the growth rate of the BW*ΔtolC* pASK*-tolC* strain on the same plate to account for plate to plate variability in absolute growth rates (Table 2 and Supplementary Figure 3). Due to the phenotype of the D371V mutant showing decreased growth rate even in the absence of a test substrate, no direct comparisons in growth rates could be made for this mutation.

**TABLE 2:** Comparison of the relative growth rates of the TolC mutants in different antibiotics.

	**Cam**	**Doc**	**Fus**	**Nal**	**Nov**	**Vanc**
BW25113 pASK-RM	154	**160**	110	123	**126**	90
BW*ΔtolC* pASK-RM	**29**	**36**	**19**	103	**7**	109
BW*ΔtolC* pASK*-tolC*	100	100	101	100	100	100
D121N	**79**	**81**	116	89	94	85
Q129L	119	104	92	101	**96**	92
N145L	77	103	117	100	89	101
R158D	84	94	95	82	**88**	**81**
V198D	76	121	92	104	81	85
N332L	89	**84**	104	134	113	**80**
N342A	94	95	92	135	104	86
Y344F	96	**89**	**75**	113	**86**	89
Q346L	102	106	88	110	109	87
S350F	**22**	**43**	**34**	**57**	**11**	118
Q352A	102	98	83	89	92	86
Q352E	100	98	105	116	106	88
YFRS	**15**	**40**	N/A	**45**	N/A	84
D371V	**9**	**27**	N/A	**33**	**76**	**63**
D374V	85	91	100	**82**	**87**	100
K383D	75	**76**	105	**62**	85	**78**
K383E	111	103	101	103	92	89
R390E	**38**	**76**	93	88	N/A	100
RENT	89	85	71	106	N/A	78

*Average maximum relative growth rates of strains expressing mutant TolC in the presence of the indicated antibiotics, using the complemented strain as the 100% reference value. Values given are averages, *n* = 5 biological replicates except for fusidic acid (*n* = 4). Bold values are statistically significantly different to the complemented strain (Welch’s *T*-test *p*-value < 0.05).*

Measured by relative growth rate, the S350F mutation increased susceptibility to all efflux substrates tested, but not to vancomycin (Table 2 and Supplementary Figure 3). The YFRS mutant grew slower than the BW*ΔtolC* pASK-*tolC* strain in the presence of each of the efflux substrates tested, though the mutation had no effect on growth rate in the presence of vancomycin. Eight of the mutations – N145L, V198D, N342A, Q346L, Q352A, Q352E, K383E, and RENT – had no effect on growth rates in the presence of any of the tested substances under the conditions tested. Significantly, all of the other mutations (D121N, Q129L, R158D, N332L, Y344F, D374V, K383D, and R390E) displayed substrate-specific effects on growth rate. The substrates with which the mutations had an effect were different for each of these mutations, indicating that the effect is not due to a global decrease in efflux activity which might have a greater effect on substrates pumped more rapidly and a lesser effect on substrates pumped more slowly. None of the three mutations that caused the MIC of vancomycin to decrease (namely Y344F, Q352A and the R390E/N392T “RENT”) caused a change in growth rate in the presence of vancomycin under the concentration tested, although the R158D, N332L, and K383D mutations did.

### Dye Efflux Assays Reveal Mutations With Diametrically Opposing Effects on Efflux

**FIGURE 3:**
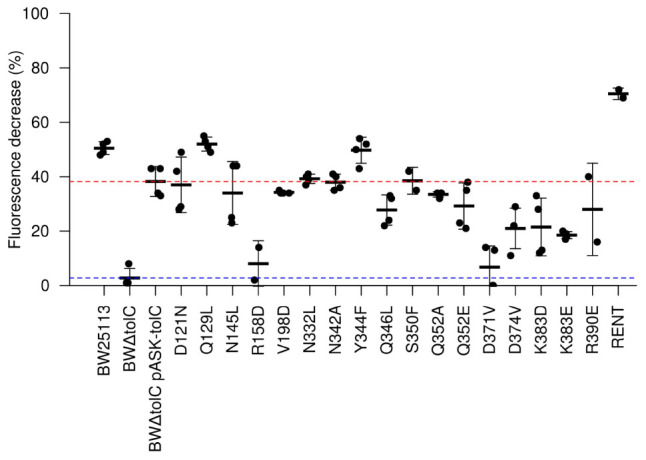
Efflux activity as determined by Nile Red efflux assays. A value of 100 would indicate complete loss of fluorescence. Each dot represents a unique biological replicate; error bars represent standard deviation. Values were normalized first to account for minor differences in OD_600_ between samples. The red and blue dotted lines represent efflux efficiency of the wild-type TolC positive control and the BW*ΔtolC* negative control, respectively. Note that R158D and D371V show efflux decrease similar to the TolC-deletion control, while “RENT” double-mutant provides statistically significant increase in efflux efficiency.

To assess efflux activity more directly, the mutations were used in dye-efflux assays using Nile Red, which fluoresces in hydrophobic environments such as cell membranes. Cells were pre-loaded by incubation with both CCCP (to abolish Δp) and Nile Red. After washing to remove excess CCCP and Nile Red, fluorescence was measured every 27 s. After five readings, glucose was injected to re-energize the cells and initiate efflux activity. Fluorescence measurements were normalized to fluorescence per unit OD_600_, to account for variations in number of cells per sample, and further normalized to a percentage of initial fluorescence, to account for variation in the starting fluorescence values. This allows determination of the percentage of fluorescence that is lost due to efflux activity for each strain (Figure 3). Two of the mutations (R158D and D371V) showed an apparently complete loss of efflux activity. Using a cut-off limit *P*-value of 0.05 as determined by Bonferroni’s pairwise *T*-test, only the K383E mutation (*P*-value = 0.018) caused a statistically significant decrease in Nile Red efflux compared to the BW*ΔtolC* pASK-*tolC* strain; any apparent decrease in efflux activity for the other mutations was not statistically significant. Unexpectedly, the RENT double mutation increased efflux activity (*P*-value = 0.00012).

### Phenotypic Effects Are Not Due to TolC Protein Levels in the Membrane

To ensure that the observed phenotypic differences were not due to significantly altered protein expression levels, TolC expression within the strains showing phenotypic differences were compared by Western blot. Membrane fractions were harvested from stationary phase cultures, solubilised and the relative total membrane protein concentration of each sample measured by Bradford assay. An equal amount of total membrane protein from each sample was resolved by SDS-PAGE, transferred to nitrocellulose and probed using polyclonal anti-TolC antibodies. Alkaline phosphatase-conjugated secondary antibodies and NBT/BCIP were used to develop the blot. The blot indicated that BW*ΔtolC* pASK-*tolC* overproduced TolC protein compared to the wild-type BW25113 pASK-RM strain (Supplementary Figure 4). Of the mutations assessed, D121N, S350F, and YFRS showed comparable levels of TolC protein as the complemented strain. The K383D and R390E mutations showed lower TolC levels than the complemented strain, but higher than the wild-type strain. The amount of D371V mutant TolC protein was substantially lower than the amount of TolC in the complemented strain, but appeared comparable to the amount of TolC protein in the wild-type strain. The slight widening of the bands visible for each strain preparation is consistent with the full-length unprocessed protein and mature protein, respectively.

## Discussion

In this study, we set out to analyze the effect of a number of point-mutants of the solvent-accessible residues of the TolC channel on the efflux activity and substrate specificity. Mutations used in this study were chosen based on structural predictions that they would either affect the AcrA-TolC interaction or impact the propensity of the TolC channel to become open, derived from the comparative analysis of the open-state (2VDD.pdb, 2VDE.pdb; Bavro et al., 2008; 2XMN.pdb; Pei et al., 2011) and closed-state (1EK9.pdb; Koronakis et al., 2000) TolC crystal structures. We also wanted to characterize the antibiotic profiles of some residues for which phenotypically pronounced mutations have been reported earlier, such as R390 (Augustus et al., 2004) and D121 (Bokma et al., 2006).

Hence the mutants introduced relate to residues which can be broadly split into the following groups by their location on the TolC α-barrel and assumed mechanism of action (see Figure 2 and Supplementary Material): Y362, R367 (tip of helix H7 and loop-connecting H7 with H8, respectively), – gating residues (aka “primary gates”) – sealing the tip of the channel; D371, D374 (H8) – gating residues (secondary gates) – restricting the lumen of the channel; D121, Q129 (H3), Q352, K383, R390 (helix7) – intra-protomer groove solvent exposed residues; V198 – equatorial domain loop; N145 (H3); R158 (H4); N332, N342, Q346 (H7) – inter-protomer groove of TolC; Y344; S350 – interprotomer interface (H7) potentially impacting inter-helical packing;

Only one of the mutations tested was not introduced purposefully – R390E/N392T (RENT) – arose spontaneously during the process of R390E mutagenesis and was included due to the possibility of it being a suppressor mutation. There is no obvious reason why this mutation spontaneously arose, or why it has a substrate-specific effect on phenotype. N392 is located at the middle section of H8, which forms part of the “moving” hairpin H7/H8, however, the location of the residue is rather far away from the dilation of the aperture as seen in the available structures, facing partway into the lumen of the TolC channel. Intriguingly, N392 has been reported to become more solvent-accessible in the presence of AcrAB (Krishnamoorthy et al., 2013), consistent with the possible rearrangement of the H7/H8 hairpin associated with the TolC channel opening upon complex assembly and AcrA interaction. While the exact mechanism of its action is difficult to rationalize at present, it is notable, that position N392 is located beneath the equatorial domain of TolC, some 30 Å from the periplasmic tip of the OMF that undergoes dilation. It is well-established that the equatorial domain is required for TolC functionality. Specifically, residues 198–214 within the equatorial domain determine which transporter-PAP pair function with the OMF (Yamanaka et al., 2007). In addition, the C-terminal part of the OMF that contributes to the discontinuous equatorial domain is also involved in regulating the OMF function. Namely, the C-terminal part of the equatorial domain is required for functionality in TolC (Yamanaka et al., 2001, 2004); while in OprM, the C-terminal 22 amino acids, which also form part of the equatorial domain, are similarly involved in determining functionality with cognate and non-cognate pumps and PAPs (Bai et al., 2010, 2014).

Consistent with the importance of the equatorial domain, several residues we mutated in addition of the aforementioned N392 exhibit phenotypic effects (D121, N332, N342, and R390) and lie in direct proximity to it. The D121N mutation had previously been described from a random mutagenesis study (Bokma et al., 2006). That study identified this mutation as enabling TolC to function with MexAB but did not investigate the impact of this mutation upon AcrAB-TolC functionality. Our results establish that the mutation D121N causes a mild but substrate-specific effect. The effect of the mutation is difficult to reconcile with a simple increase in the open-state probability of the TolC channel given the differential antibiotic-specificity, which should have been the same across the spectrum of substrates if that was the case. One possible explanation for the observed effect is that TolC residue D121, which is close to the equatorial domain and on the external face of TolC channel, may discriminate between cognate and non-cognate PAP partner proteins. While this could be achieved directly through interaction with the PAP-hairpin in the adaptor wrapping model of interaction, it is unclear how this function would be achieved in the tip-to-tip model. At the same time, despite the same considerations applying to the mutation V198D that is located on the equatorial domain loop, this mutant did not exhibit any detectable difference in MICs against the WT TolC under complementation conditions tested.

Although current models of pump function do not attribute any role for TolC in substrate selection or vetting, it is apparent from our MIC and growth kinetic data that some of the mutations tested in this study have an effect for only a subset of antibiotics and dyes, changing the substrate profile of the efflux pump complex. This could be for a variety of reasons. At positions that line the TolC lumen (Q129, Y344, and D374; see Figure 2), this effect may arise due to changing the electrostatic and hydrophobicity profiles of the environment through which a substrate must pass and even direct steric hindrance, hence affecting the kinetics of substrate passage through the channel. The same explanation could be at least true for residues lining the periplasmic tip of TolC (e.g., N145) – although mutations at the tip region have previously been reported to decrease the affinity of TolC for its partner proteins (Tikhonova et al., 2011). However, it seems highly unlikely that any interaction between the channel and substrate is also the cause of substrate-specific effects for mutations at positions mapping to the outside of the channel (D121, R158, N332, and R390), as the substrate should not directly pass through or interact with these positions. Some PAP-OMF interactions have been observed to be naturally substrate-specific, notably those involving MexJ (Chuanchuen et al., 2002). The MexJK PAP-transporter pair utilizes either OprM or OpmH dependent upon the efflux substrate (Chuanchuen et al., 2005). While there is no suggestion of the possibility of the OMF-swap in the current study, changes of the state of the TolC channel could potentially be sensed by the PAPs, and communicated to the RND pump, which in turn may translate into functional differences. Indeed, in the RND metal-ion efflux systems such as ZneCAB and CusCFBA, the PAP component is involved in loading of the substrate into the transporter protein (Bagai et al., 2008; De Angelis et al., 2010). While such function has not been reported for PAPs in multidrug RND-systems, it may explain how mutations on the external face of the TolC alpha-barrel might have substrate-specific effects. A change in the interface presented to the cognate PAP could alter the conformation of the PAP during its binding either of substrate or TolC, and thereby exclude certain substrates from being passed to the transporter within tripartite complex. Given the location of these mutations on the surface of the alpha-barrel (Figure 2), such communication would likely necessitate an interaction between AcrA-hairpin similar to the “adaptor wrapping” deep-interpenetration model of tripartite assembly (Figure 1). However, the latest structural data, utilizing cryo-tomography to assess the AcrAB-TolC complex in its native cellular *trans-*envelope state, found evidence of complexes only in the tip-to-tip conformation (Shi et al., 2019).

The aspartate residues at TolC positions 371 and 374 form two stacked rings of negative charge, which act as a cation trap and form the tightest constriction within the TolC lumen (Andersen et al., 2002b). The lower of these, D371, may be involved in stabilizing the open state of TolC upon rearrangement of the ionic network at the periplasmic tip of TolC, namely by forming a new interaction with R367 (Bavro et al., 2008). This may partially explain why the R367A mutation causes hyper-susceptibility to efflux substrates (Augustus et al., 2004) even though removal of the charge at this position does not on its own cause constitutive dilation of the TolC channel (Pei et al., 2011).

Importantly, the overall effect on the cell of the D371V mutation was not simply a result of loss of TolC function, but had some additive effect. The deletion of *tolC* is well tolerated in laboratory conditions, while the D371V mutation was not. It is likely that the mutated protein is incapable of fulfilling its normal role in removal of toxic metabolic products (Ruiz and Levy, 2014), a task presumably fulfilled by other proteins in the *tolC*-deleted strain (Rosner and Martin, 2009). Previously reported mutation of the aspartate ring D371 was to alanine, the small-side chain of which does not protrude far into the TolC lumen, therefore alleviating any steric and charge restrictions imposed by the aspartate in that position. Indeed, the D371A mutation was reported not to impact upon either deoxycholic acid resistance or haemolysin export (Andersen et al., 2002b). Mutation to valine (D371V) reported here, while superficially similar is expected to introduce a new rigid steric barrier at the narrowest part of the channel due to the beta-branching of the Val side-chain. Hence, this mutation was predicted to cause disruption of the electrostatic interactions and H-bond networks at the periplasmic tip, due to the introduction of the new bulky hydrophobic ring, impacting the existing negatively charged D374 ring just one helical-turn above in the TolC lumen. Significantly, modeling the position of the valine sidechains onto open state structures available suggests that there are severe steric constraints imposed on them, while they pack tightly with the aliphatic portion of the R367 in the closed state, without requiring any notable conformational changes from the closed structure. It is therefore plausible that the D371V mutation caused a loss of function by stabilizing the closed-state of TolC.

In summary, this work indicates that single-substitution mutations located within, or in close proximity to the equatorial-domain of TolC can have a negative impact on TolC-function. This impact does not appear to stem from effects on TolC expression levels and folding, as it is substrate-specific. These substrate-specific effects also indicate that the inner-membrane transporter/PAP-protein pair is not the sole determinant of substrate specificity within the tripartite efflux-assemblies, but significantly, our results hint toward a possible new role for the OMF proteins in substrate selection and vetting during efflux events.

At present, these findings can’t be straightforwardly reconciled with the “tip-to-tip” model of PAP-OMF interaction that is favored by the recent cryo-EM structural studies, and while it may be possible that other modes of PAP-OMF interaction are required at different stages of the efflux process, it is clear that further investigation is needed to clarify the exact mechanisms involved.

## Data Availability Statement

The data supporting the findings of this study are available in the article and Supplementary Materials. The whole datasets (including raw data) are available from the corresponding author on reasonable request.

## Author Contributions

VB and RM conceived and designed the experiments, analyzed the data, and wrote the manuscript. RM performed the experiments. Both authors contributed to the article and approved the submitted version.

## Conflict of Interest

The authors declare that the research was conducted in the absence of any commercial or financial relationships that could be construed as a potential conflict of interest.
