# Supplementary material for: Mutations in the TolC Periplasmic Domain Affect Substrate Specificity of the AcrAB-TolC Pump
Source: Front Mol Biosci. 2020 Jul 21;7:166. doi: 10.3389/fmolb.2020.00166 (PMC7396618; doi:10.3389/fmolb.2020.00166)
Supplement: Supplementary file 1 [file Data_Sheet_1.pdf]

Supplementary Information

Construction of expression vector pASK-RM

The commercially-available vector pASK-IBA13plus was mutated first using the pASK_altNde_F and pASK_altNde_R primer pair, to remove the NdeI site within the *tetR* gene encoding the tetracycline repressor. To introduce a new NdeI site at the start codon driven by the tetracycline promoter, primers pASK_NdeI_F and pASK_NdeI_R were used in a second round of mutagenesis. Finally, to introduce a new STOP site immediately downstream of the XhoI site used for cloning, so that only the Leu-Glu encoded in the XhoI site would be expressed, the pASK_Stop_F and pASK_Stop_R primer pair were used in a the third round of mutagenesis.

Supplementary table 1. Primers used for generation of pASK-RM from pASK-IBA13plus.

Primer name	Primer sequence
pASK_altNde_F	5'-GCCTTCTTATTCGGCCTTGAATTGATCATCTGCGGATTAGAAAAA-3'
pASK_altNde_R	5'-TTTTTCTAATCCGCAGATGATCAATTCAAGGCCGAATAAGAAGGC-3'
pASK_NdeI_F	5'-TAACTTTAAGAAGGAGATATACATATGTGGAGCCACCCGCAGTTCGA-3'
pASK_NdeI_R	5'-TCGAACTGCGGGTGGCTCCACATATGTATATCTCCTTCTTAAAGTTA-3'
pASK_Stop_F	5'-CGGGGATCCCTCGAGTGAGACCTGCAGGGGGAC-3'
pASK_Stop_R	5'-GTCCCCCTGCAGGTCTCACTCGAGGGATCCCCG-3'

Methodology for computational analysis to inform mutagenesis

JANUS is a programme designed to determine positions in a protein sequence that make up part of the active site of an enzyme (Addington et al., 2013) by analysing residue conservation patterns and identifying co-variant pairs of residues. While TolC is not an enzyme, and therefore does not have an active site as such, we employed JANUS based on the idea that the PAP-interaction interface will be subject to similar conservation selectin pressure where PAPs might be considered as the functionally important part of the protein. The programme calculates importance from a series of sequence alignments, each of which must be similar proteins that have a different substrate and each of which must be based upon a structural alignment. The conservation at each position for each alignment is

used to determine whether or not a position is important for that family of proteins – if a position is variable in proteins that perform the same function with the same substrate, then that position is highly unlikely to be important for the function of the protein, although this is calculated based on similarity, not identity. Next, the positions are compared between alignments – if a position shows conservation in both sets of sequences, then it is not responsible for determining specificity, regardless of whether or not it is important for function. The positions that are conserved only within the respective alignments, but not when comparing the two sets of alignments, are potentially important for determining specificity, and the scores for these positions are therefore calculated based upon an algorithm taking into account differences in size, charge and hydrophobicity between the two alignments.

All full-length sequences available in the UniProt database matching {gene: tolC AND organism: coli}, {(gene: tolC OR oprM) AND organism: Pseudomonas}, {gene: tolC AND organism: Vibrio} and {(gene: tolC OR mtrE) AND organism: Neisseria} were aligned using Clustal Omega. This alignment was then separated into four smaller alignments, one for each of *E. coli*, *Pseudomonas spp.*, *Vibrio spp.* and *Neisseria spp.*, keeping the same alignment positions as generated by Clustal Omega. The *E. coli* alignment was used as input file 1, with each of the other files used individually as input file 2. The output files were analysed to determine likely importance of positions only at or further from the outer membrane than the equatorial domain, as positions closer to the outer membrane are unlikely to be involved in an interaction with the cognate PAP. Positions at which the side chain faces into the lumen were also disregarded from this analysis, as they are also unlikely to contact the PAP.

Results from JANUS were used in conjunction with Consurf (Ashkenazy et al., 2010), an online server which automatically generates sequence alignments based on a single pdb code as the input, scores the conservation at each position, and maps the categorised conservation score to the input pdb file (Supplementary Figure 1). Partial sequences and duplicate entries were excluded from the alignment using the manual selection feature. Positions that showed limited variability were taken to

be those with potential to determine specificity of interaction with the PAP. Those that had a very low conservation score (hypervariable positions) are clearly not under selective pressure and were assumed not to be critical to the function, while we reasoned that those which are absolutely conserved are likely to be required for structural integrity of the protein reasons and are unlikely to be tolerated, so they were not selected for use in this study.

Rationale for TolC mutations used in this study

For a brief overview of the following text, see Supplementary Table 1.

D121N - This mutation has previously been shown to adapt TolC to function with MexAB (Bokma et al., 2006), but the reason behind this adaptation, and any effect of this mutation on the AcrAB-TolC complex have not been shown. Position 121 is close to the equatorial domain, and may interact with R390, an interaction which, if broken by AcrA, would weaken the inter-helical interactions that hold TolC in its closed conformation. The objectives for introducing this mutation were therefore to determine firstly whether the previously reported gain-of-function with MexAB is caused by constitutive opening of the TolC channel, and secondly whether the mutation has any additional, unreported effects on AcrAB-TolC function.

Q129L – Although this residue faces into the TolC lumen, in the closed state of the channel it is within interaction-distance from the N381 on H8, which may result in stabilisation of the closed state; while on the other hand, modelling suggests that upon relaxation of the coiled coils, this residue may contribute to the intra-protomer groove formed at the surface between H3 and H8 (Janganan et al., 2011a). By mutation to leucine, a bulky, beta-branched hydrophobic residue, the tight packing of the coiled coils may be compromised, potentially affecting both aperture and AcrA interaction. JANUS gives a high importance score (combined score of 397 when compared to MtrE, VceC and OprM) for this position, suggesting that it is likely to be involved in determining selection of the PAP.

N145L – Mutation to cysteine at this position cross-links quite poorly to AcrB, compared to mutation of other positions in the same turn between H3/H4 of TolC (Tamura et al., 2005). The poor cross-

linking upon mutation of residue at this position was previously interpreted to be indicative that it is involved in the interaction with AcrB, and that the mutation causes sufficient loss of interaction between the proteins for efficient cross-linking to occur, possibly by affecting stability or proximity of the residues within the complex. The distances between the three N145 positions within the TolC trimer also match well with the distances between the Q255 and D256 on the AcrB trimer, and superimposing the TolC tip to the β-hairpins of AcrB suggest that these positions may well interact, although this would rotate TolC by 60° about an axis perpendicular to the membrane compared to the previously proposed AcrB-TolC interaction (Bavro et al., 2008). While current models of tripartite pump assembly do not favour direct contact of AcrB and TolC (see **Figure 1**), we wanted to verify whether this mutation may have any detectable effects on the efflux.

R158D – Mutation of this position to cysteine has been shown to be unable to cross-link to AcrA even when the long cross-linking agent LC-SPDP (with a 15.7 Å linker arm) was used (Lobedanz et al., 2007). Other positions in the same region of the protein do enable cross-linking when mutated to cysteine, which may indicate that this particular mutation prevents the TolC-AcrA interaction from being stable enough for cross-linking to take place. This position may also be involved in hydrogen bonding with Q346 and S350, as part of the inter-protomer interaction, disruption of which by binding of AcrA could facilitate outward movement of helices, dilating the channel. Even if not involved in channel dilation, this region of the external face of TolC contains a particularly high local concentration of charged residues, matching charges of which could be used as “discriminator” residues for PAP-OMF interaction. Under such hypothesis, switching the charge on one of these “discriminator” residues should make the PAP-OMF pair incompatible and could disrupt binding of a partner protein. This mutation was therefore expected to cause loss of efflux activity and possibly to also increase channel dilation.

V198D – Located on the flexible equatorial domain loop connecting the two structural repeats of TolC, this position would be a less obvious candidate for mutation were it not for the reported gain-of-function with MexAB (Bokma et al., 2006). This gain of function of TolC with MexAB has not

been comprehensively investigated however, namely the reason for this gain of function, and any possible impact upon functionality with AcrAB remaining unknown. It is notable however that several prominent charged residues lie in the vicinity of the V198 in the loop (namely D121, Y125, K383 and R390). Change of the character of the residue at this position may cause electrostatic repulsion with the underlying charged residues and/or may impact the fold of the equatorial domain, which may adopt a different conformation, in turn possibly affecting the interaction with AcrA hairpin, compromising the optimal packing of TolC and AcrA hairpin tips which in turn is expected to affect efflux.

N332L – This residue is located on H7, just below the equatorial domain. It forms part of a network of hydrophilic residues, including N173, D176, N177, E180 and R328, at an inter-protomer interface at the position where the α-barrel helical trajectories begin to twist, stabilising the kink in H7 that helps to generate the closed conformation. Disruption of the interactions in this area by AcrA binding could be expected therefore to lead to channel opening. In other proteins, the equivalent position is alanine in MtrE and OprM, but asparagine in VceC. Given that VceAB is capable of functioning with TolC (Vediyappan et al., 2006), but neither MtrC nor MexA are capable of functioning with TolC without mutational adaptation, this position could potentially play the role of one of the "discriminator" residues that play the role of determinants for PAP selection. By placing a bulky hydrophobic leucine at the position described, this mutation was hypothesised to impact the packing of the inter-protomer H7/H3 groove and impact the probability of open state of the channel, thus likely having an impact on the efflux.

N342A – Based on our computational analysis, this position was found to be hypervariable in OMFs from different species, but is well conserved within a species. Furthermore, this residue is located on H7 in the second-structural repeat of the TolC protein, with the equivalent position in H3 in the first structural repeat of the protein being glutamine, a conservative substitution, which may allow similar docking of the PAP-hairpins with a 3:6 protomer arrangement under the "deep-interpenetration" scenario (Janganan et al., 2011b). This indicates that the position could be essential for the interaction

with partner PAPs. The JANUS score at this position is a mid-range score, as the combined score to each of the other proteins used in the comparison is 193 (ranked 233 out of 428), suggesting that there may be limited requirement for mutation at this position to switch binding partners. In the equivalent position in the other proteins analysed by JANUS, MtrE (aspartate) and VceC (arginine) have opposite charges to each other, while OprM contains glutamine. AcrA and OprM are known to interact in a non-functional manner, thus suggesting that this position may be required for the interaction but not for the functionality of the two proteins. To verify whether conservation of this residue may be critical under such scenarios we included it into the list of mutants to be tested.

Y344F – In the partially open state of TolC exemplified by 2VDD structure (Bavro et al., 2008), Y344 appears to interact with T120 and N167, forming an inter-protomer interaction in the inside of the channel. However, in the 1EK9 crystal structure, the slight helical rearrangement of H7 appears to cause a disruption of this interaction. The position is conserved in VceC (also tyrosine) but not in either MtrE or OprM (leucine and glutamine, respectively), and although due to its location it is not likely to be directly involved in PAP selection it may influence it indirectly by affecting the geometry of the inter-protomer groove. Results from Consurf indicate that this position is quite well conserved (group 9), in a region which is otherwise largely poorly conserved but not hypervariable (groups 5 and 6). Consistent with this JANUS gives a mid-level score to this position (88 to MtrE and 97 to OprM), indicating that although possibly important in selection of the partner protein, it is not likely to be as important as other residues discussed above. Y344F mutation was picked at this position as to prevent possible hydrogen bonds from forming, thus influencing the open/closed probability of the channel, but the conservative change is deliberately selected as it is unlikely to cause major disruption to the folding and trimerisation of TolC.

Q346L – Mutation of the equivalent position in MtrE (K397) has previously been shown to increase sensitivity to tetracycline and erythromycin, but not other antibiotics (Janganan et al., 2011a; Janganan et al., 2011b). The score from Consurf (group 9) indicates that this position is well conserved across many OMF channel proteins, and manual analysis of multiple sequence alignments

indicates that this is indeed the case, except for in OprJ, which is only known to function with MexCD, although MexCD-OprM is also functional when OprM is overexpressed. Glutamine at this position is conserved in VceC but not in MtrE or OprM (lysine and aspartic acid, respectively); the charged residues of MtrE and OprM, which are not capable of complementing TolC (or vice versa), suggests that this position may be partially responsible for restricting functionality, but not binding, of PAPs with TolC. The JANUS scores reflect this, giving a high score for the comparison with MtrE (192) and a medium score (78) for comparison with OprM. Analysis of the TolC structure indicates that this position interacts with R158 and D162, both of which are also highly conserved, in an inter-protomer interaction. Channel opening and helical rearrangement associated with PAP binding may therefore be expected to disrupt this interaction. Mutation to leucine was reasoned to eliminate side-chain hydrogen bonding at this position, possibly impacting the PAP-OMF interaction and functionality with AcrA.

S350F – Initially described as a mutation impacting the trimerisation and membrane insertion of TolC, and correspondingly causing hypersensitivity phenotypes (Werner et al., 2003; Augustus et al., 2004;) the actual mechanism of action of this mutant remains unclear. This position is part of an inter-protomer (H7-H4’) interaction network involving the H4’ residues D162 and R158, and introduction of a bulky side chain such as phenylalanine is expected to result in steric clashes affecting inter-helical packing, which should translate into pronounced phenotypic effects. We also decided to pursue this mutation to clarify the literature and determine to what extent it impacts the TolC protein levels in the membrane. Intriguingly, serine is conserved at this position in OpmH and OprJ, while an alanine residue is conserved at the equivalent positions in MdsC, OprM, OprN and MtrE. MdsA can function with either TolC or MdsC (Song et al., 2014), while AcrA is only capable of functioning with TolC. Similarly, MexC functions with either OprJ or OprM (Gotoh et al., 1998), and MexJ functions with either OprM or OpmH dependent upon the substrate (Chuanchuen et al., 2005), while MexA is only capable of functioning with OprM. It is tempting to speculate that these conservation patterns may indicate that the hydroxyl-group may be required for full functionality with a specific subset of PAP

partners, while other PAPs can function irrespective of whether the hydroxyl group is present, although such investigation lies beyond the remit of the current study.

Q352A/E – Despite being only 17 Å from the periplasmic tip of TolC which is supposed to engage the AcrA, mutation of this position to cysteine does not facilitate cross-linking to AcrA even when using a 15.7 Å linker arm (Lobedanz et al., 2007), although S124C mutation, located further up the coiled-coil interface 31 Å away from the periplasmic tip facilitated cross-linking with a shorter linker arm. This may indicate that Q352 is essential for the binding of AcrA to TolC, and mutation prevents this interaction from being sufficiently stable to facilitate cross-linking. Consurf indicates that while this position is quite variable between proteins, closer analysis of sequence alignments reveals that when glutamine is not conserved at this position, it is substituted by a charged residue possibly suggesting that potential some charge-specific "discriminator" interaction could take place in certain OMF-PAP pairs at such position similar to suggestions of Stegmeier et al., 2007. This interpretation is also consistent with the high JANUS scores for this position (a combined score of 384), indicating that it may be required in selection of partner proteins. Therefore this position was assessed by two mutations - the first, Q352A, aiming to disrupt any potential polar/charged interactions at this position between AcrA and TolC, while the second, Q352E, a more subtle probe was aimed at charge-dependency at this position without changing any other parameters such as side-chain length.

Y362F/R367S ("YFRS") – This double mutation is known to disrupt the hydrogen- and ionic-bonding network at the periplasmic tip of TolC, causing slightly wider dilation of the channel (as defined across the D374 narrowing of the channel) than the similar "YFRE" mutation (Bavro et al., 2008; Pei et al., 2011). While the "YFRE" mutation has been shown to be more susceptible to novobiocin (an efflux substrate) and to have lower binding affinity to both AcrA and AcrB (Tikhonova et al., 2011), there have been no reports of the effects of the YFRS mutation on efflux activity or binding of partner proteins, hence we added this it to this study.

D371V – As mentioned in the previously, TolC has two concentric aspartate rings formed by Asp-residues located on H8 at the periplasmic end of its lumen, which form the narrowest constriction of

its channel and act as ion-selective gates (Andersen et al., 2002). This position has previously been mutated to alanine as part of a double mutation, along with D374A, which caused a loss of cation selectivity, and subsequently, greater permeability of the channel. Upon complete channel dilation, the outward movement of the H7/H8 helical pair may bring D371 into contact with R143, forming an ion-bridge that is speculated to stabilise the open state. While the D371V mutation is similar to D371A tested before, it introduces a beta-branched hydrophobic residue which is predicted to cause not only a significant occlusion, but also a restrictive hydrophobic barrier. In addition, it was expected that during opening of the channel the side-chains of Val may interfere with the packing of the helices, restricting full opening.

D374V – As with the D371V mutant, above, this position forms the second aspartate ring that acts as an ion-selective gate (Andersen et al., 2002), also known as “secondary gate” (Bavro et al., 2008) the dilation of which is required for passage of higher-molecular weight cargoes, including antibiotics. Similarly to the D371V mutation, the D374V mutation was chosen as the change of the electrostatic properties at the selectivity gate is expected to impact the efflux of the cargoes. It is also notable, that in the structure of VceC, which has the equivalent of D371/V374 (Federici et al., 2005), this selectivity gate is stable and appears closed in the available structure, suggesting that the D374V mutation should not impact trimer stability.

K383D/E – The K383D mutation has previously been reported to cause increased sensitivity to novobiocin (an effluxed substrate), but not to vancomycin, suggesting that the increase in novobiocin sensitivity is not caused by a substantial increase of influx through the TolC channel (Bavro et al., 2008). This sensitivity was reversed by a compensatory mutation in AcrA (D149K) mapping to the hairpin-domain of AcrA, suggesting that a direct interaction may take place between AcrA and TolC at this location (Bavro et al., 2008). Similarly, a charge reversal of the equivalent position in MtrE (E434) caused increased vancomycin sensitivity only in the presence of the cognate PAP, indicating that the mutation itself does not cause constitutive opening of the channel (Janganan et al., 2013), and reinforcing the possibility of direct PAP-OMF interaction at this position. The K383D was therefore

included for more detailed antibiotic sensitivity and efflux-profile analysis, while the K383E was included to determine whether the deleterious effect is caused only by the charge reversal or whether the length of the side chain may also be a factor.

R390E(/N392T) – Random mutagenesis of TolC yielded the R390C mutation which caused hypersensitivity to all tested drugs (efflux-substrates and vancomycin), suggesting that the apparent loss of function may at least in part be attributable to increased influx of the efflux substrates through TolC, although decreased efflux activity may also contribute (Augustus et al., 2004). In both TolC and at the equivalent position in MtrE, mutation of this position to glutamate has been shown to increase vancomycin susceptibility (Augustus et al., 2004; Bavro et al., 2008; Janganan et al., 2011b). The R390E mutation was therefore included to study in greater detail the effect of this mutation, particularly in a possible interaction with AcrA. The R390E/N392T "RENT" double mutant was isolated following mutagenesis, and was found to have spontaneously arisen on the transformation plate, it was included to test whether it had arisen as a suppressor mutation.

Table 1: The mutations used in this study.

Mutation	Location relative to the alpha-barrel of TolC	Helical turns from periplasmic tip based on 1EK9.pdb	Structural rationale	Additional comments
D121N	External on H3, close to equatorial domain	7	Interacts with R390; disruption of the interaction may relax the coiled coils resulting in dilation of the TolC channel	Adaptive gain-of-function mutation, produces an active chimeric pump with MexAB[a]
Q129L	Internal on H3	4	Interacts with N381 on H8; change in local hydrophobicity between closed and open structures	Inferred from computational data
N145L	External, periplasmic tip	0	Likely to be an interactive residue providing contact with either AcrA (tip-to-tip model) or AcrB (deep interpenetration model)	Cysteine mutation cross-links poorly to AcrB compared to mutation of other positions in the same H3/H4 turn[b]
R158D	External on H4	2	H-bonds with Q346 and S350 as part of intra-protomer interaction; high local concentration of hydrophilic residues	Mutation to cysteine does not result in crosslinking of the PAP (AcrA) even when using a 15.7 Å linker arm[c]
V198D	External, on the equatorial domain loop	N/A	A hydrophobic residue in an otherwise very hydrophilic local environment;	Adaptive gain-of-function mutation, produces an active chimeric pump with MexAB[a]
N332L	External on H7, at the level of the equatorial domain	9	Part of a network of hydrophilic residues, including N173, D176, N177, E180 and R328, at the inter-protomer interface	Lack of conservation in homologues that do not complement loss of *tolC*[d]<br>Conserved within the internal structural repeats of TolC
N342A	External on H7, underneath the equatorial domain	6	Surface exposed, may interact with the equatorial domain	Inferred from computational data
Y344F	Internal on H7	5	Interacts with T120 and N167, forming an inter-protomer interaction.	Inferred from computational data
Q346L	External on H7	5	Interacts with R158 and D162 in an inter-protomer interaction	Mutation of the equivalent position in MtrE (K397) increased sensitivity to substrates[e,f]<br>Inferred from computational data[s]
S350F	External on H7	4	Part of an inter-protomer hydrogen- and ionic-bonding network	Conflicting information in the literature[g,h]<br>Conservation and cross-functionality in homologues[i,j,k]
Q352(A/E)	External on H7	3	-	Mutation to cysteine did not facilitate cross-linking to AcrA even when using a 15.7 Å linker arm[c]<br>Computational data

Y362F / R367S (YFRS)	External at the periplasmic tip	0	Interact to seal the periplasmic tip[m,n]	Mutation decreased binding affinity to partner proteins[p]
D371V	Internal on H8	1	Upon dilation, D371 may interact with R143, to stabilise the open state<br>Mutation to a hydrophobic residue may disrupt either the open or closed states due to electrostatic incompatibility	Ion-selective gate, mutation to alanine causes loss of ion co-ordination[q]
D374V	Internal on H8	2	Mutation to a hydrophobic residue may disrupt either the open or closed states due to electrostatic incompatibility	Ion-selective gate, mutation to alanine caused loss of ion co-ordination[q]<br>VceC is stable and closed (like TolC) but has valine at this position[r]
K383(D/E)	External on H8	4	Previous reports[m], suggested that it may directly interact with the PAP, namely D149.	The K383D mutation increased sensitivity to novobiocin, but not by a substantial increase of influx through the TolC channel[m]<br>Charge reversal of the equivalent position in MtrE (E434) caused increased vancomycin sensitivity only in the presence of the PAP[f]
R390E	External on H8, close to the equatorial domain	6	Interacts with D121; disruption of the interaction may relax the coiled coils resulting in dilation of the TolC channel	In both TolC and at the equivalent position in MtrE, mutation to glutamate increased vancomycin susceptibility[f,m]<br>R390C mutation caused hypersensitivity to all tested efflux substrates and vancomycin[g]

[a] - Bokma et al., 2006; [b] - Tamura et al., 2005; [c] - Lobedanz et al., 2007; [d] - Vediyappan et al., 2006; [e] - Janganan et al., 2011a; [f] - Janganan et al., 2011b; [g] - Augustus et al., 2004; [h] - Werner et al., 2003; [i] - Chuanchuen et al., 2005; [j] - Gotoh et al., 1998; [k] - Song et al., 2014; [m] - Bavro et al., 2008; [n] - Pei et al., 2011; [p] - Tikhonova et al., 2011; [q] - Andersen et al., 2002b; [r] - Federici et al., 2005

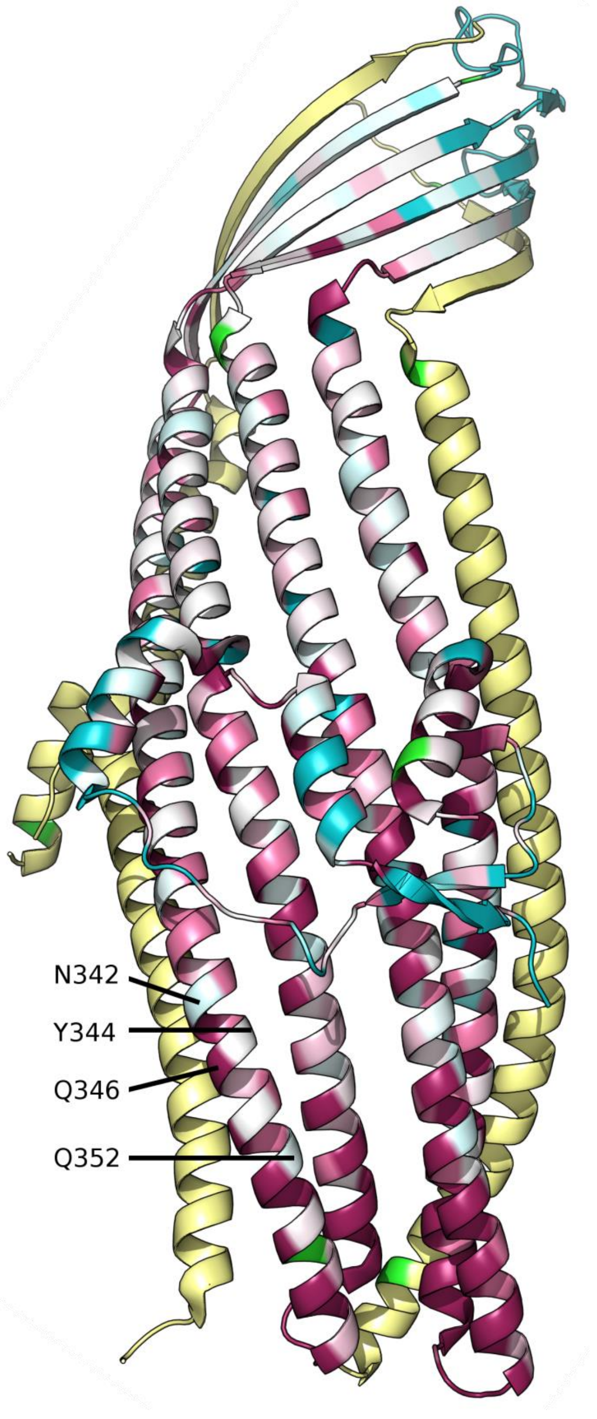

Supplementary Figure 1. Consurf output of conservation in OMF sequences. Using the 1EK9 structure as the query, the Consurf results were mapped directly onto a single protomer in the same structure. In pale yellow are adjacent helices belonging to the neighboring protomers. Each position is coloured on a scale from blue through white to purple, with blue being hypervariable, white being semi-variable and purple being most conserved. Positions in green lacked sufficient data in the alignment to calculate a conservation score. The Consurf result contributed to selection for mutation of the labelled positions.

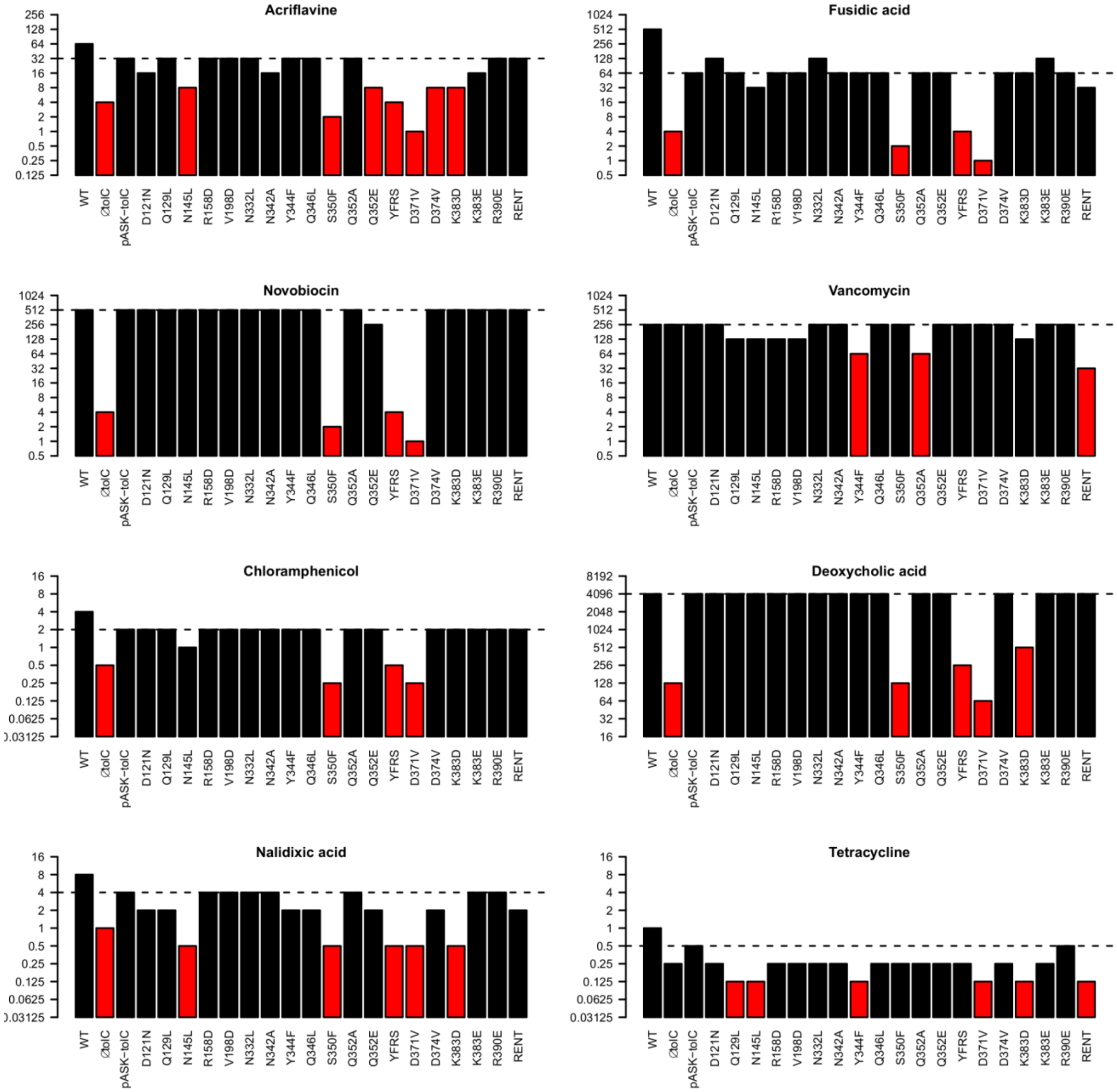

Supplementary Figure 2. Visual representation of the MIC data from Table 1 for different mutants arranged by antibiotic. Significant decrease of MIC (difference ≥ 2 doubling dilutions) is represented in red bars.

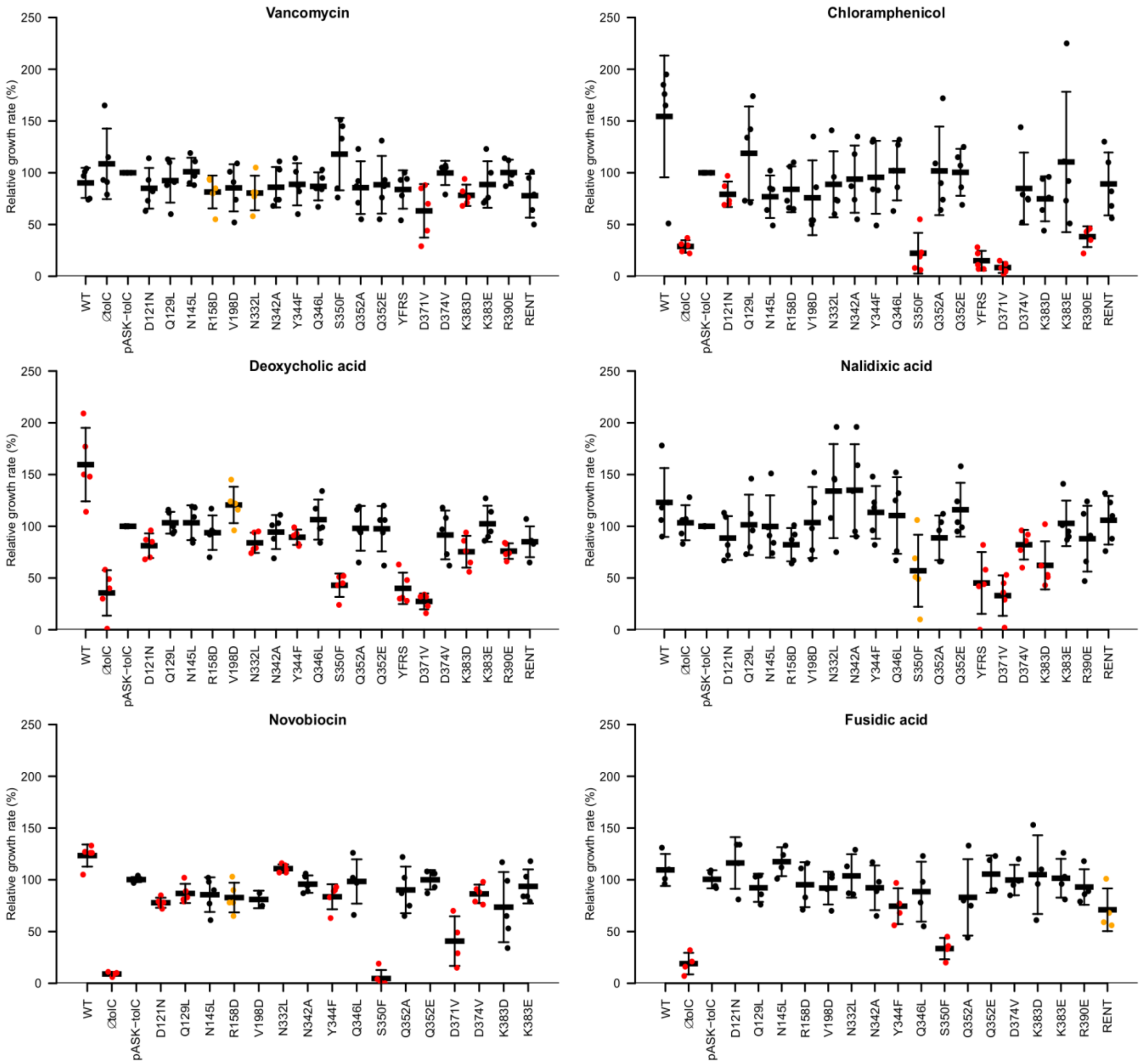

Supplementary Figure 3. Relative growth rates of *tolC* mutants in the presence of antibiotics at sub-inhibitory concentrations.

The relative growth rate is the maximum growth rate expressed as a percentage of the maximum growth rate of the complemented strain in the same conditions. WT - BW25113 pASK-RM; ΔtolC – BW25113 *ΔtolC* pASK-RM; pASK-tolC – BW25113 *ΔtolC* pASK-*tolC*(WT). Red: p-value < 0.05. Orange: p-value < 0.06. Each dot represents a unique biological replicate; error bars represent standard deviation.

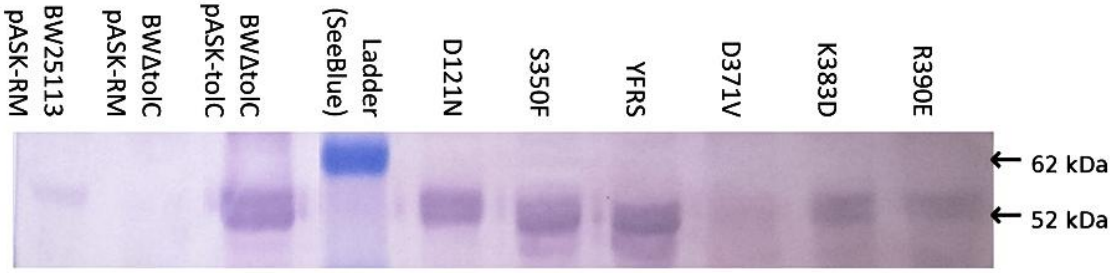

Supplementary Figure 4. Comparison of the TolC protein levels in the membrane for the different mutants tested. Western blot showing TolC protein (52 kDa) for mutants of *tolC* grown in the same conditions as in preparation for flow cytometry. The sample volume loaded for SDS-PAGE was adjusted to load equal amounts of total membrane protein per sample. The 62 kDa MW marker band is visible in blue.

References for Supplementary information

Addington, T.A., Mertz, R.W., Siegel, J.B., Thompson, J.M., Fisher, A.J., Filkov, V., et al. (2013) Janus: Prediction and ranking of mutations required for functional interconversion of enzymes. *Journal of Molecular Biology,* 425 1378-1389. doi: 10.1016/j.jmb.2013.01.034.

Andersen, C., Koronakis, E., Hughes, C. and Koronakis, V. (2002) An aspartate ring at the TolC tunnel entrance determines ion selectivity and presents a target for blocking by large cations. *Molecular Microbiology,* 44 1131-1139. doi: 10.1046/j.1365-2958.2002.02898.x.

Ashkenazy, H., Erez, E., Martz, E., Pupko, T. and Ben-Tal, N. (2010) ConSurf 2010: Calculating evolutionary conservation in sequence and structure of proteins and nucleic acids. *Nucleic acids research,* 38 W529-W533. doi: 10.1093/nar/gkq399.

Augustus, A., Celaya, T., Husain, F., Humbard, M. and Misra, R. (2004) Antibiotic-sensitive TolC mutants and their suppressors. *Journal of Bacteriology,* 186 1851-1860. doi: 10.1128/JB.186.6.1851-1860.2004.

Bavro, V.N., Pietras, Z., Furnham, N., Perez-Cano, L., Fernandez-Recio, J., Pei, X.Y., et al. (2008) Assembly and channel opening in a bacterial drug efflux machine. *Molecular cell,* 30 114-121. doi: 10.1016/j.molcel.2008.02.015.

Bokma, E., Koronakis, E., Lobedanz, S., Hughes, C. and Koronakis, V. (2006) Directed evolution of a bacterial efflux pump: Adaptation of the *E. coli* TolC exit duct to the *Pseudomonas* MexAB translocase. *FEBS letters,* 580 5339-5343 . doi: 10.1016/j.febslet.2006.09.005.

Chuanchuen, R., Murata, T., Gotoh, N. and Schweizer, H. (2005) Substrate-dependent utilization of OprM or OpmH by the *Pseudomonas aeruginosa* MexJK efflux pump. *Antimicrobial Agents and Chemotherapy,* 49 2133-2136. doi: 10.1128/AAC.49.5.2133-2136.2005.

Federici, L., Du, D., Walas, F., Matsumura, H., Fernandez-Recio, J., McKeegan, et al. (2005) The crystal structure of the outer membrane protein VceC from the bacterial pathogen *Vibrio cholerae* at 1.8 angstrom resolution. *Journal of Biological Chemistry,* 280 15307-15314. doi: 10.1074/jbc.M500401200.

Gotoh, N., Tsujimoto, H., Nomura, A., Okamoto, K., Tsuda, M. and Nishino, T. (1998) Functional replacement of OprJ by OprM in the MexCD-OprJ multidrug efflux system of *Pseudomonas aeruginosa*. *FEMS microbiology letters,* 165 21-27. doi: 10.1111/j.1574-6968.1998.tb13122.x.

Janganan, T.K., Bavro, V.N., Zhang, L., Matak-Vinkovic, D., Barrera, N.P., Venien-Bryan, C., et al. (2011a) Evidence for the assembly of a bacterial tripartite multidrug pump with a stoichiometry of 3:6:3. *Journal of Biological Chemistry,* 286 26900-26912. doi: 10.1074/jbc.M111.246595.

Janganan, T.K., Zhang, L., Bavro, V.N., Matak-Vinkovic, D., Barrera, N.P., Burton, M.F., et al. (2011b) Opening of the outer membrane protein channel in tripartite efflux pumps is induced by interaction with the membrane fusion partner. *Journal of Biological Chemistry,* 286 5484-5493. doi: 10.1074/jbc.M110.187658.

Janganan, T.K., Bavro, V.N., Zhang, L., Borges-Walmsley M.I., Walmsley A.R. (2013) Tripartite efflux pumps: energy is required for dissociation, but not assembly or opening of the outer membrane channel of the pump. Molecular Microbiology, 88 (3):590-602 doi: 10.1111/mmi.12211

Lobedanz, S., Bokma, E., Symmons, M.F., Koronakis, E., Hughes, C. and Koronakis, V. (2007) A periplasmic coiled-coil interface underlying ToIC recruitment and the assembly of bacterial drug efflux pumps. *Proceedings of the National Academy of Sciences of the United States of America,* 104 4612-4617. doi: 10.1073/pnas.0610160104.

Pei, X., Hinchliffe, P., Symmons, M.F., Koronakis, E., Benz, R., Hughes, C. and Koronakis, V. (2011) Structures of sequential open states in a symmetrical opening transition of the TolC exit duct. *Proceedings of the National Academy of Sciences of the United States of America,* 108 2112-2117. doi: 10.1073/pnas.1012588108.

Song, S., Hwang, S., Lee, S., Ha, N. and Lee, K. (2014) Interaction mediated by the putative tip regions of MdsA and MdsC in the formation of a *Salmonella*-specific tripartite efflux pump. *Plos One,* 9 e100881. doi: 10.1371/journal.pone.0100881.

Stegmeier, J.F., Polleichtner, G., Brandes, N., Hotz, C. and Andersen, C. (2006) Importance of the adaptor (membrane fusion) protein hairpin domain for the functionality of multidrug efflux pumps. Biochemistry, 45 10303-10312. doi: 10.1021/bi060320g.

Tamura, N., Murakami, S., Oyama, Y., Ishiguro, M. and Yamaguchi, A. (2005) Direct interaction of multidrug efflux transporter AcrB and outer membrane channel TolC detected via site-directed disulfide cross-linking. *Biochemistry,* 44 11115-11121. doi: 10.1021/bi050452u.

Tikhonova, E.B., Yamada, Y. and Zgurskaya, H.I. (2011) Sequential mechanism of assembly of multidrug efflux pump AcrAB-ToIC. *Chemistry & biology,* 18 454-463. doi: 10.1016/j.chembiol.2011.02.011.

Vediyappan, G., Borisova, T. and Fralick, J.A. (2006) Isolation and characterization of VceC gain-of-function mutants that can function with the AcrAB multiple-drug-resistant efflux pump of *Escherichia coli*. *Journal of Bacteriology,* 188 3757-3762. doi: 10.1128/JB.00038-06.

Werner, J., Augustus, A. and Misra, R. (2003) Assembly of TolC, a structurally unique and multifunctional outer membrane protein of *Escherichia coli* K-12. *Journal of Bacteriology,* 185 6540-6547. doi: 10.1128/JB.185.22.6540-6547.2003.
